# Therapeutic vaccine-mediated Gag-specific CD8^+^ T-cell induction under anti-retroviral therapy augments anti-virus efficacy of CD8^+^ cells in simian immunodeficiency virus-infected macaques

**DOI:** 10.1038/s41598-020-68267-w

**Published:** 2020-07-09

**Authors:** Midori Nakamura-Hoshi, Yusuke Takahara, Saori Matsuoka, Hiroshi Ishii, Sayuri Seki, Takushi Nomura, Hiroyuki Yamamoto, Hiromi Sakawaki, Tomoyuki Miura, Tsuyoshi Tokusumi, Tsugumine Shu, Tetsuro Matano

**Affiliations:** 1 AIDS Research Center, National Institute of Infectious Diseases, 1-23-1 Toyama, Shinjuku-ku, Tokyo, 162-8640 Japan; 2 The Institute of Medical Science, The University of Tokyo, 4-6-1 Shirokanedai, Minato-ku, Tokyo, 108-8639 Japan; 3 Institute for Frontier Life and Medical Sciences, Kyoto University, 53 Kawahara-cho, Shogoin, Sakyo-ku, Kyoto, 606-8507 Japan; 4 ID Pharma Co., Ltd., 6 Ohkubo, Tsukuba, Ibaraki 300-2611 Japan

**Keywords:** Immunology, Microbiology, Molecular biology

## Abstract

Anti-retroviral therapy (ART) can inhibit HIV proliferation but not achieve virus eradication from HIV-infected individuals. Under ART-based HIV control, virus-specific CD8^+^ T-cell responses are often reduced. Here, we investigated the impact of therapeutic vaccination inducing virus-specific CD8^+^ T-cell responses under ART on viral control in a macaque AIDS model. Twelve rhesus macaques received ART from week 12 to 32 after simian immunodeficiency virus (SIV) infection. Six of them were vaccinated with Sendai virus vectors expressing SIV Gag and Vif at weeks 26 and 32, and Gag/Vif-specific CD8^+^ T-cell responses were enhanced and became predominant. All macaques controlled viremia during ART but showed viremia rebound after ART cessation. Analysis of in vitro CD8^+^ cell ability to suppress replication of autologous lymphocytes-derived SIVs found augmentation of anti-SIV efficacy of CD8^+^ cells after vaccination. In the vaccinated animals, the anti-SIV efficacy of CD8^+^ cells at week 34 was correlated positively with Gag-specific CD8^+^ T-cell frequencies and inversely with rebound viral loads at week 34. These results indicate that Gag-specific CD8^+^ T-cell induction by therapeutic vaccination can augment anti-virus efficacy of CD8^+^ cells, which may be insufficient for functional cure but contribute to more stable viral control under ART.

## Introduction

HIV induces persistent infection leading to AIDS onset in humans. Anti-retroviral therapy (ART) can control HIV replication and prevent AIDS progression. However, ART is unable to eradicate viruses and HIV-infected individuals need to receive life-long therapy for AIDS prevention^1–4^. It has been suggested that ART does not always exhibit complete shutdown of viral replication, while blips of undetectable levels of viral replication under ART may cause chronic persistent inflammation, possibly leading to progression of aging-related diseases^5–9^.

Virus-specific cytotoxic CD8^+^ T-cell responses play a central role in the control of HIV and simian immunodeficiency virus (SIV) replication^10–14^. Because ART-based HIV control is often accompanied by reduced virus-specific CD8^+^ T-cell responses^15,16^, augmentation of effective CD8^+^ T-cell responses by therapeutic vaccination may contribute to more complete shutdown of viral replication under ART, although therapeutic vaccine trials under ART have failed to achieve functional HIV cure, sustained viral control after ART cessation^17–20^. Optimization of vaccine immunogens is important for induction of effective CD8^+^ T-cell responses. Cumulative studies have indicated strong anti-HIV efficacy of Gag-specific CD8^+^ T cells^14,21–26^. Furthermore, Vif-specific CD8^+^ T cells have recently been indicated to exert strong suppressive pressure against HIV replication^27,28^. However, the impact of therapeutic vaccination inducing these CD8^+^ T cells under ART have not fully been determined.

We have previously developed Sendai virus (SeV) vectors expressing SIV/HIV antigens and have shown the potential of these vectors to induce viral antigen-specific T-cell responses in macaques and humans^29–31^. In the present study, SIVmac239-infected rhesus macaques were immunized with SeV vectors expressing SIVmac239 Gag and Vif antigens under ART. This immunization enhanced Gag/Vif-specific CD8^+^ T-cell responses. Our analysis indicated association of Gag-specific CD8^+^ T-cell responses with anti-SIV efficacy of CD8^+^ cells post-vaccination.

## Results

### Analysis of plasma viral loads

**Figure 1:**
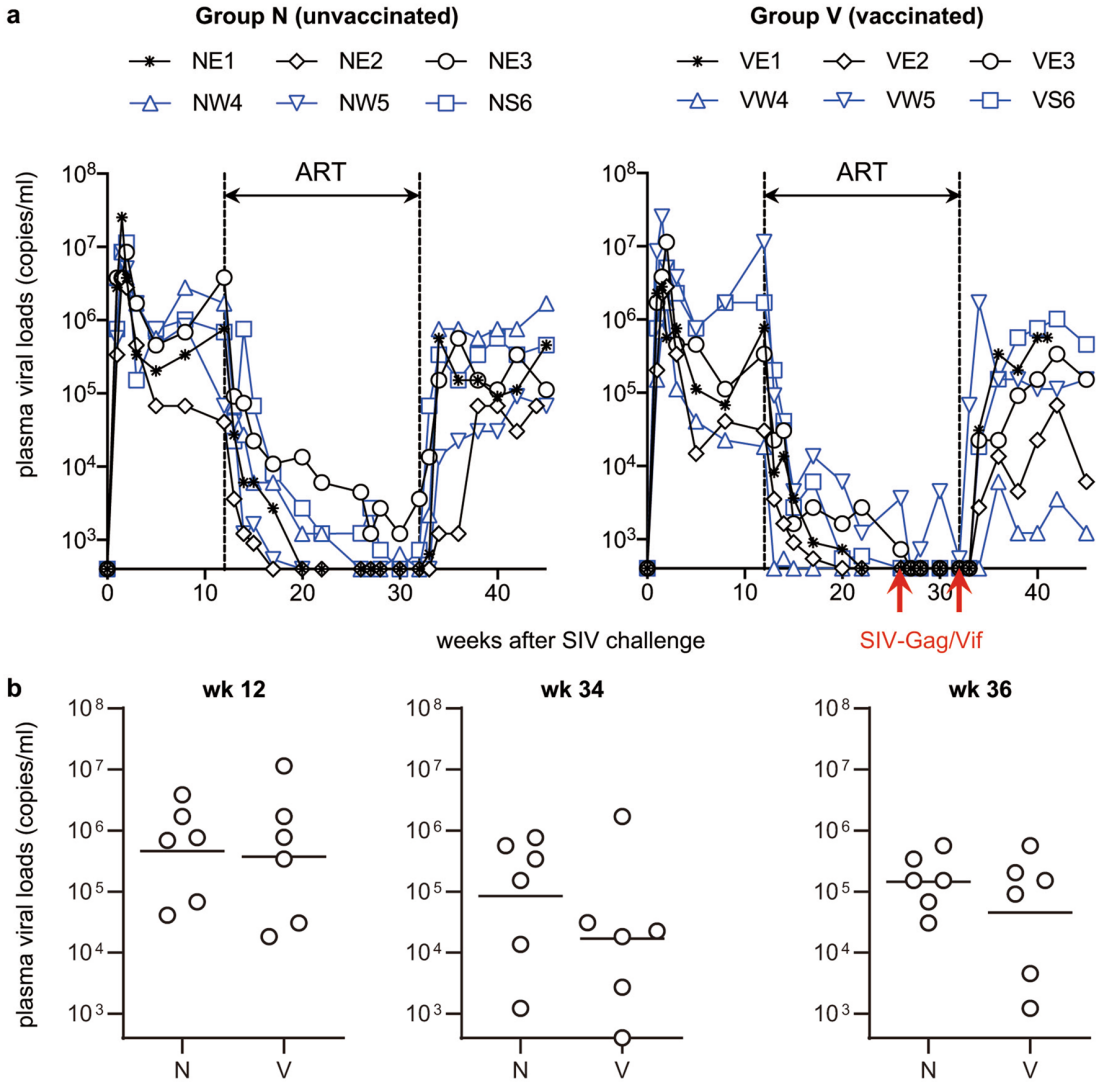
Plasma viral loads after SIVmac239 infection. (**a**) Viral loads (SIV *gag* RNA copies/ml plasma) determined as described previously^30^. The lower limit of detection is approximately 4 × 10^2^ copies/ml. Six Group N (left panel) and six Group V (right panel) macaques received ART from week 12 to 32 after SIVmac239 infection. Group V macaques received therapeutic SeV-Gag/Vif vaccination at weeks 26 and 32 post-infection. (**b**) Comparison of viral loads at weeks 12, 34 and 36 post-infection between Groups N and V. No significant difference was observed.

Twelve Burmese rhesus macaques consisting of six animals sharing the MHC-I haplotype *90–010-Ie* (E), four sharing *89–075-Iw* (W) and two sharing *91–010-Is* (S) were used in the present study as shown in Table 1. These macaques were intravenously inoculated with SIVmac239 and received ART from week 12 to 32 post-infection. All the macaques showed persistent viremia after the SIV infection and reduced plasma viral loads after ART initiation at week 12 (Fig. 1). These macaques were divided into two groups, Group N (n = 6) receiving no vaccination and Group V (n = 6) receiving vaccination at weeks 26 and 32 post-infection. The Group V macaques consisting of three E-positive, two W-positive and one S-positive were intranasally immunized with Gag- and Vif-expressing SeV vectors at weeks 26 and 32 post-infection. Two of the Group N (NE3 and NW5) and all the six Group V animals were intravenously administered with polyclonal anti-SIV immunoglobulin G (anti-SIV IgG). After ART cessation at week 32, all macaques showed reappearance of plasma viremia. No clear difference was observed in viral loads post-ART between two anti-SIV IgG-treated and four untreated macaques in Group N. While two Group V macaques VE2 and VW4 exhibited relatively lower viral loads post-ART, no significant difference in viral loads post-ART was observed between Groups N and V.

**Table 1:** Macaque experimental protocol.

Group	Experiment	Macaque	MHC-I haplotype	SIV_mac239_ iv^a^	ART^b^	SeV-Gag/Vif in^c^	a-SIV IgG iv^d^
N	Exp 1	NE1	E	Wk 0	Wk 12–32	(−)	(−)
N	Exp 1	NE2	E	Wk 0	Wk 12–32	(−)	(−)
N	Exp 2	NE3	E	Wk 0	Wk 12–32	(−)	Wk 32
N	Exp 1	NW4	W	Wk 0	Wk 12–32	(−)	(−)
N	Exp 2	NW5	W	Wk 0	Wk 12–32	(−)	Wk 32
N	Exp 1	NS6	S	Wk 0	Wk 12–32	(−)	(−)
V	Exp 1	VE1	E	Wk 0	Wk 12–32	Wks 26 and 32	Wk 32
V	Exp 1	VE2	E	Wk 0	Wk 12–32	Wks 26 and 32	Wk 32
V	Exp 2	VE3	E	Wk 0	Wk 12–32	Wks 26 and 32	Wk 32
V	Exp 1	VW4	W	Wk 0	Wk 12–32	Wks 26 and 32	Wk 32
V	Exp 2	VW5	W	Wk 0	Wk 12–32	Wks 26 and 32	Wk 32
V	Exp 1	VS6	S	Wk 0	Wk 12–32	Wks 26 and 32	Wk 32

^a^Animals were intravenously inoculated with SIVmac239.

^b^Animals received ART from week 12 to 32 post-infection.

^c^Animals were intranasally immunized with SeV-Gag and SeV-Vif.

^d^Animals were intravenously administered with anti-SIV IgG.

### Analysis of antigen-specific CD8^+^ T-cell responses

**Figure 2:**
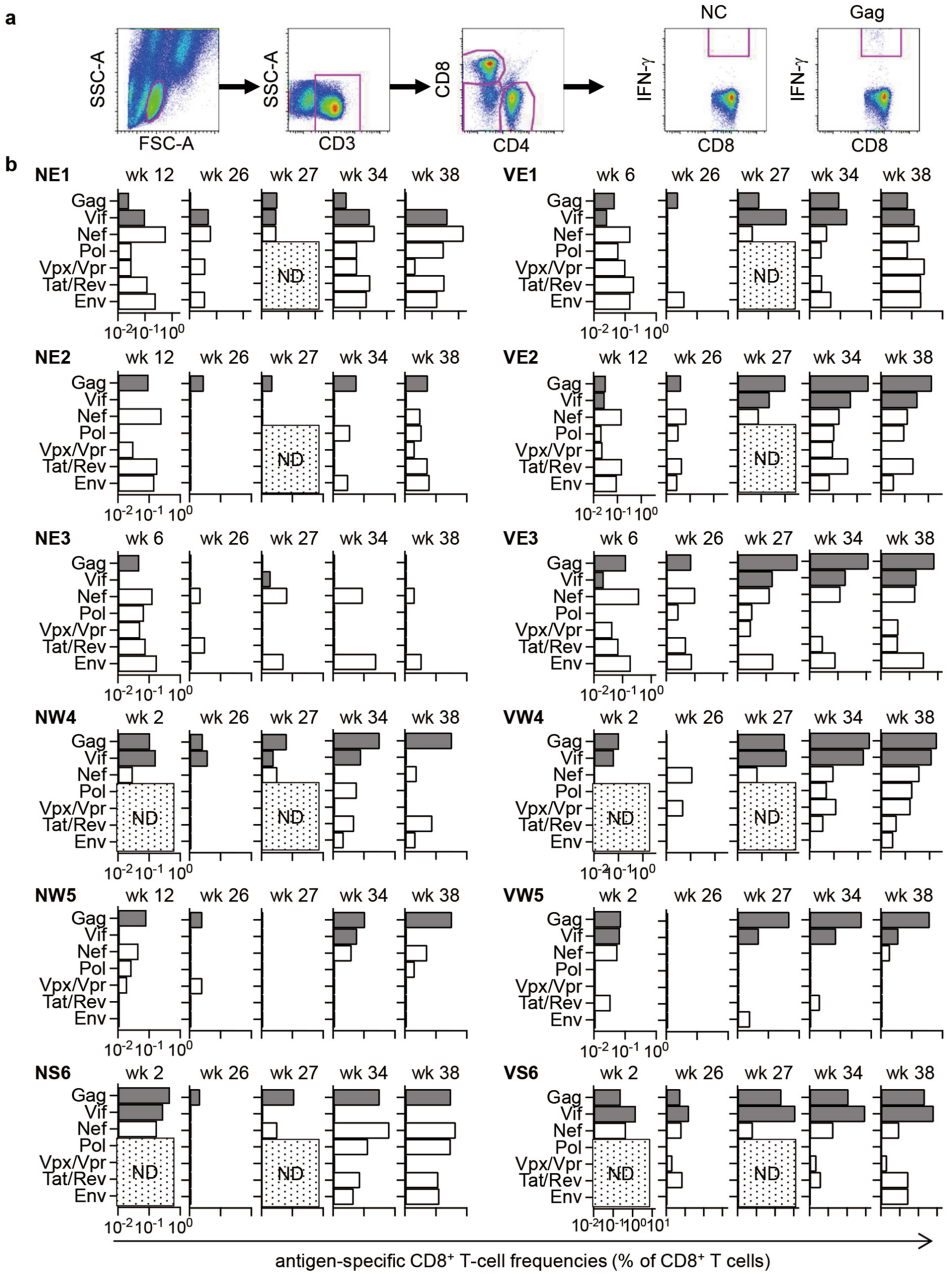
SIV antigen-specific CD8^+^ T-cell responses after SIVmac239 infection. (**a**) Representative gating schema for detection of specific IFN-γ induction after peptide stimulation in flow cytometric analysis. Data on PBMCs of macaque VW4 at week 38 without stimulation (NC) and with stimulation using overlapping peptides spanning the N-terminal half of Gag proteins (Gag) are shown. (**b**) SIV antigen-specific CD8^+^ T-cell frequencies at indicated time points after SIVmac239 infection. CD8^+^ T-cell responses targeting SIV Gag, Vif, Nef, Pol, Vpx/Vpr, Tat/Rev, and Env were examined by detection of specific IFN-γ induction after stimulation using overlapping peptides spanning individual antigens. Log-transformed CD8^+^ T-cell frequencies are shown. PBMCs were obtained at weeks 2–12 (before ART initiation), 26 (just before the 1st vaccination), 27 (1 week after the 1st vaccination), 34 (2 weeks after ART cessation) and 38 post-infection and subjected to the analyses. *ND* not determined because of the limitation of available samples.

**Figure 3:**
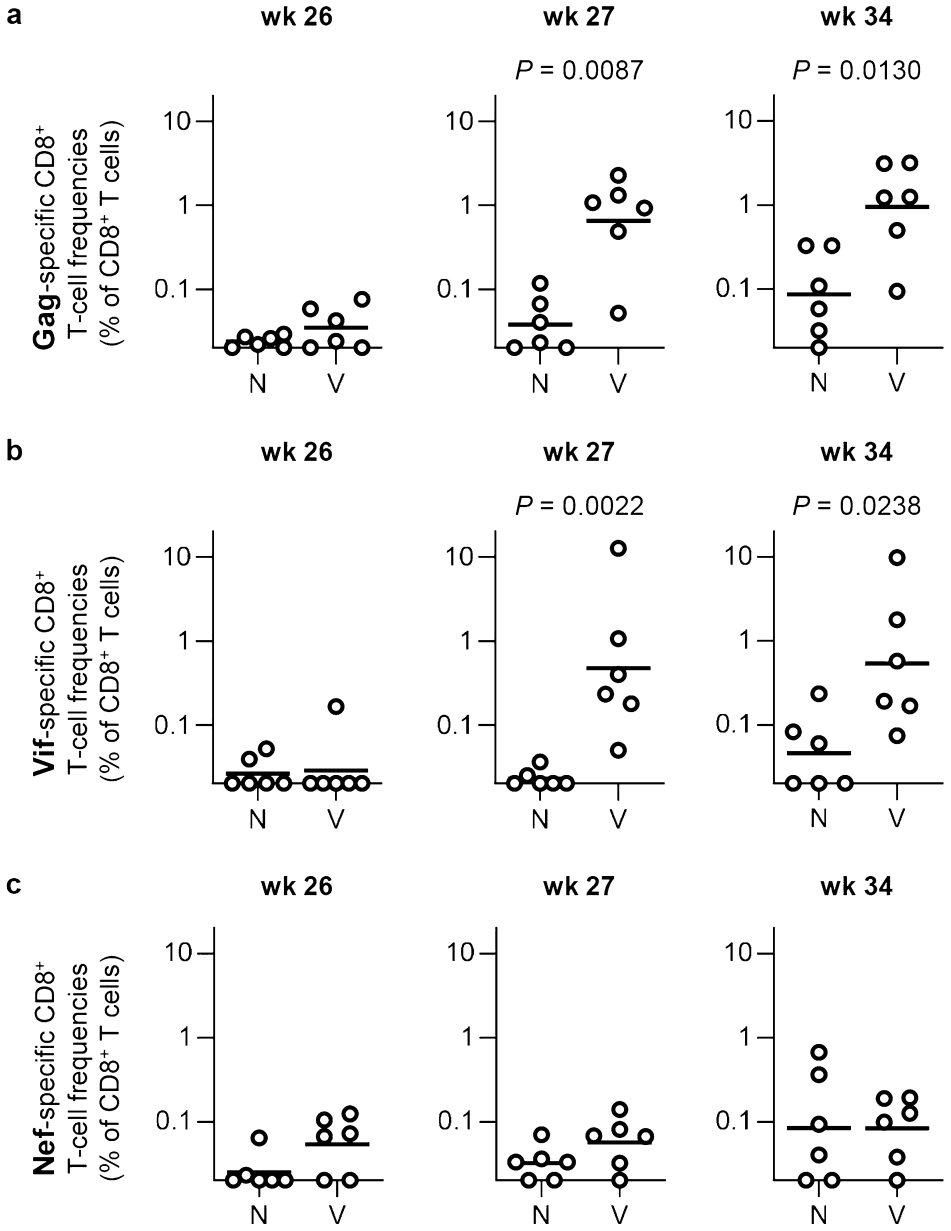
Comparison of Gag/Vif/Nef-specific CD8^+^ T-cell responses between Groups N and V. (**a**) Comparison of Gag-specific CD8^+^ T-cell frequencies between Groups N and V at weeks 26, 27, and 34 post-infection. Group V showed significantly higher frequencies than Group N at weeks 27 (*P* = 0.0087 by Mann–Whitney *U* test) and 34 (*P* = 0.0130). (**b**) Comparison of Vif-specific CD8^+^ T-cell frequencies between Groups N and V at weeks 26, 27, and 34 post-infection. Group V showed significantly higher frequencies than Group N at weeks 27 (*P* = 0.0022) and 34 (*P* = 0.0238). (**c**) Comparison of Nef-specific CD8^+^ T-cell frequencies between Groups N and V at weeks 26, 27, and 34 post-infection. No significant difference was observed.

We examined CD8^+^ T-cell responses specific for SIV individual antigens in these macaques before ART initiation, during ART, and after ART cessation by detection of antigen-specific interferon-γ (IFN-γ) induction (Fig. 2). Before the ART initiation at week 12, macaques possessing the MHC-I haplotype E showed predominant induction of Nef-, Tat/Rev- and Env-specific CD8^+^ T-cell responses, whereas those possessing the haplotypes W/S predominantly induced Gag/Vif-specific CD8^+^ T-cell responses. At week 26 during ART, these antigen-specific CD8^+^ T-cell responses were reduced as expected. All the vaccinated Group V macaques showed induction and/or enhancement of Gag/Vif-specific CD8^+^ T-cell responses at week 27 post-infection, one week after the first SeV-Gag/Vif vaccination. After the second SeV-Gag/Vif vaccination and the ART cessation at week 32, SIV antigen-specific CD8^+^ T-cell responses were enhanced. Comparison revealed significantly higher Gag- and Vif-specific CD8^+^ T-cell responses in Group V than in Group N at weeks 27 and 34, whereas no significant difference was observed before vaccination (at week 26) (Fig. 3a,b). There was no significant difference in CD8^+^ T-cell responses targeting Nef that was not included in the vaccine antigens at week 26, 27 or 34 between Groups N and V (Fig. 3c).

The unvaccinated Group N animals showed similar patterns of immunodominance pre-ART and post-ART; i.e., Nef- and Env-specific CD8^+^ T-cell responses were predominant post-ART in the E-positive unvaccinated macaques (NE1, NE2 and NE3), while Gag/Vif-specific CD8^+^ T-cell responses were predominant in the W/S-positive (NW4, NW5 and NS6). In contrast, different patterns of immunodominance were shown pre-ART and post-ART in E-positive vaccinated animals; i.e., Gag/Vif-specific CD8^+^ T-cell responses were predominant post-ART (at week 34) in the E-positive vaccinated macaques (VE1, VE2 and VE3) as well as in the W/S-positive (VW4, VW5 and VS6) in Group V. These results indicate that the SeV-Gag/Vif vaccination under ART can enhance not only dominant Gag/Vif-specific CD8^+^ T-cell responses but also subdominant ones resulting in a change in the CD8^+^ T-cell immunodominance.

### Analysis of in vitro anti-SIV efficacy of CD8^+^ cells

**Figure 4:**
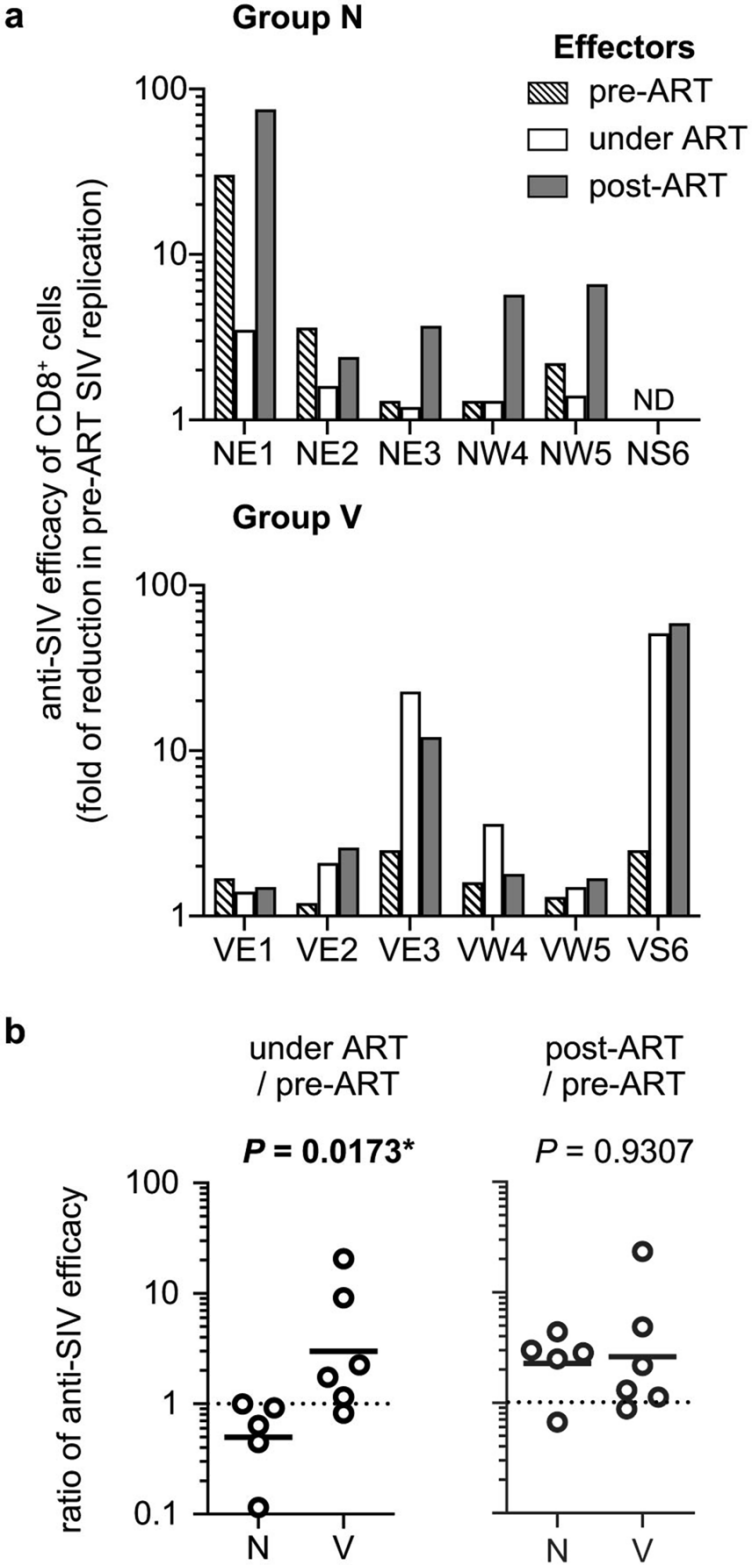
In vitro efficacy of CD8^+^ cells against pre-ART PBMC-derived SIV replication. (**a**) In vitro ability of CD8^+^ cells to suppress replication of SIVs derived from autologous pre-ART PBMCs in Groups N and V. CD8^−^ cells (target) derived from PBMCs at week 10 were cocultured with autologous CD8^+^ cells (effector) derived from PBMCs at week 10 (pre-ART), 27 (under ART), or 34 (post-ART), and the culture supernatants were subjected to analysis of SIV p27 concentrations by ELISA. The ratios of p27 concentrations in the supernatants from the coculture to those without CD8^+^ cells are shown as fold of reduction in replication of pre-ART PBMC-derived SIVs (in vitro anti-pre-ART SIV CD8^+^ cell efficacy). ND, not determined because of the limitation of sample availability. (**b**) Comparison of the ratios of anti-pre-ART SIV efficacy of CD8^+^ cells under ART (left panel) or post-ART (right panel) to pre-ART between Groups N and V. Group V showed significantly higher ratio of anti-pre-ART SIV efficacy of CD8^+^ cells under ART to pre-ART than Group N (*P* = 0.0173 by Mann–Whitney *U* test).

We performed in vitro viral suppression assay to examine the ability of CD8^+^ cells pre-ART, during ART and post-ART to suppress replication of SIVs derived from autologous peripheral blood mononuclear cells (PBMCs) pre-ART (referred to as pre-ART SIVs). The ability of CD8^+^ cells to suppress replication of SIV derived from pre-ART PBMCs (anti-pre-ART SIV CD8^+^ cell efficacy) was examined by using pre-ART CD8^-^ cells obtained at week 10 post-infection from individual animals as the target cells. CD8^+^ cells derived from PBMCs at weeks 10, 27 (1 week after the first vaccination in Group V) and 34 were prepared as effector cells pre-ART, under ART and post-ART, respectively, and reductions in SIV production from the target cells by coculture with autologous CD8^+^ cells pre-ART, under ART and post-ART were assessed, respectively (Fig. 4a). The ability of CD8^+^ cells under ART to suppress replication of pre-ART SIVs was marginal and/or reduced compared to CD8^+^ cells pre-ART in Group N but enhanced in all the Group V except for one animal (VE1). The ratio of anti-pre-ART SIV efficacy of CD8^+^ cells under ART to pre-ART was significantly higher in Group V than Group N (*P* = 0.0173 by Mann–Whitney *U* test) (Fig. 4b), implying that the therapeutic SeV-Gag/Vif vaccination can augment in vitro anti-SIV efficacy of CD8^+^ cells. The ability of CD8^+^ cells post-ART to suppress replication of pre-ART SIVs was much higher than that under ART in all the Group N animals, and there was no significant difference in the ratio of anti-pre-ART SIV efficacy of CD8^+^ cells post-ART to pre-ART between Groups N and V.

**Figure 5:**
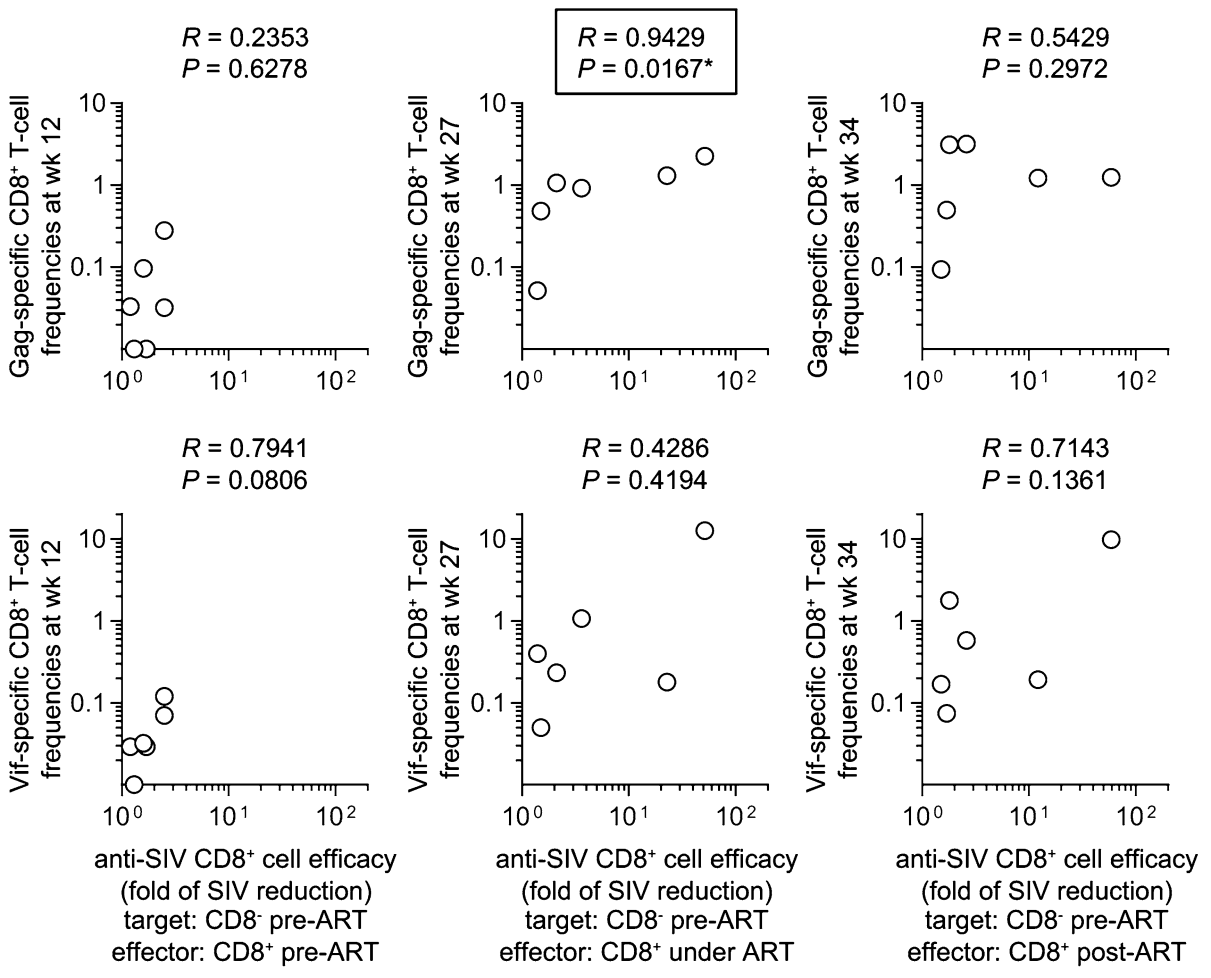
Correlation analyses of anti-SIV CD8^+^ cell efficacy against pre-ART PBMC-derived SIVs with Gag/Vif-specific CD8^+^ T-cell responses in the vaccinated animals. Correlation analysis between anti-SIV efficacy of CD8^+^ cells pre-ART (left panels), under ART (middle panels) or post-ART (right panels) against pre-ART PBMC-derived SIVs and Gag-specific (upper panels) or Vif-specific (lower panels) CD8^+^ T-cell frequencies at week 12 (left panels), 27 (middle panels) or 34 (right panels) post-infection was performed.

Next, correlation analyses were performed between in vitro anti-pre-ART SIV efficacy of CD8^+^ cells and antigen-specific CD8^+^ T-cell responses in the Group V animals. Anti-SIV efficacy of CD8^+^ cells pre-ART (at week 10) showed no correlation with Gag-specific nor Vif-specific CD8^+^ T-cell responses at week 10 post-infection (Fig. 5). Anti-pre-ART SIV efficacy of CD8^+^ cells under ART (at week 27) was significantly correlated with Gag-specific CD8^+^ T-cell responses (*R* = 0.9429, *P* = 0.0167 by Spearman test) but not with Vif-specific CD8^+^ T-cell responses at week 27 post-infection (1 week after the first vaccination) (Fig. 5). This indicates that induction of Gag-specific CD8^+^ T-cell responses by vaccination under ART can result in augmentation of the ability of CD8^+^ cells to suppress replication of pre-ART SIVs. However, the anti-pre-ART SIV efficacy of CD8^+^ cells post-ART showed no significant correlation with Gag-specific nor Vif-specific CD8^+^ T-cell responses at week 27 post-infection (Fig. 5).

**Figure 6:**
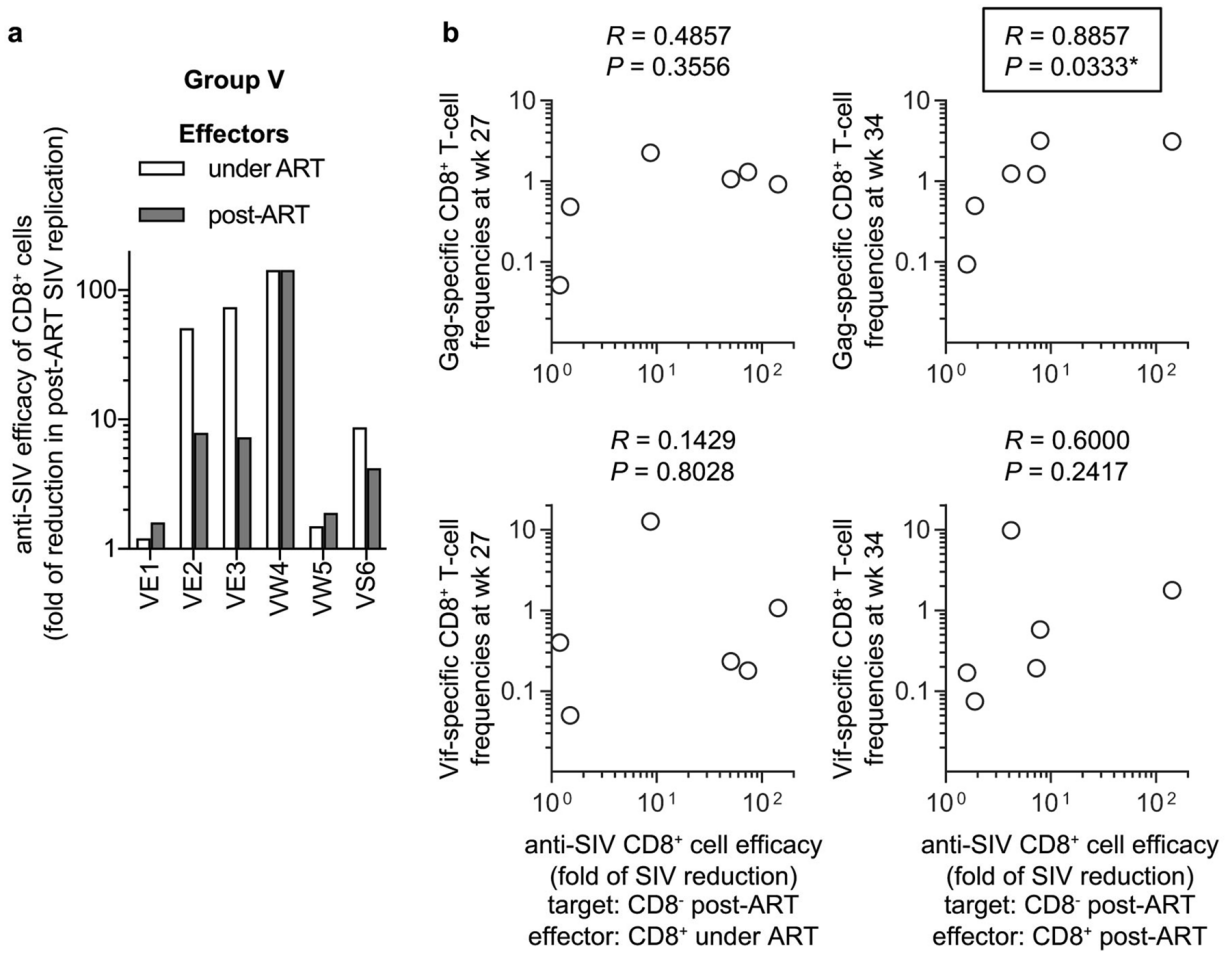
In vitro efficacy of CD8^+^ cells against post-ART PBMC-derived SIV replication. (**a**) In vitro ability of CD8^+^ cells to suppress replication of SIVs derived from autologous post-ART PBMCs in Group V. CD8^-^ cells (target) derived from PBMCs at week 34 were cocultured with autologous CD8^+^ cells (effector) derived from PBMCs at week 27 (under ART) or 34 (post-ART), and the culture supernatants were subjected to analysis of SIV p27 concentrations by ELISA. The ratios of p27 concentrations in the supernatants from the coculture to those without CD8^+^ cells are shown as fold of reduction in replication of post-ART PBMC-derived SIV (in vitro anti-post-ART SIV CD8^+^ cell efficacy). (**b**) Correlation analysis between anti-post-ART SIV efficacy of CD8^+^ cells under ART (left panels) or post-ART (right panels) and Gag-specific (upper panels) or Vif-specific (lower panels) CD8^+^ T-cell frequencies at week 27 (left panels) or 34 (right panels) post-infection.

Then, the ability of CD8^+^ cells to suppress replication of SIVs derived from autologous PBMCs post-ART (referred to as post-ART SIV) was examined by using post-ART CD8^-^ cells obtained at week 34 post-infection from individual Group V animals as the target cells (Fig. 6a). The ability of CD8^+^ cells under ART to suppress replication of post-ART SIVs (anti-post-ART SIV efficacy of CD8^+^ cells under ART) showed no significant correlation with Gag-specific nor Vif-specific CD8^+^ T-cell responses at week 27 post-infection (Fig. 6b). Instead, anti-post-ART SIV efficacy of CD8^+^ cells post-ART exhibited a significant correlation with Gag-specific CD8^+^ T-cell responses (*R* = 0.8857, *P* = 0.0333) but not with Vif-specific CD8^+^ T-cell responses at week 34 post-infection (2 weeks after ART cessation) (Fig. 6b).

**Figure 7:**
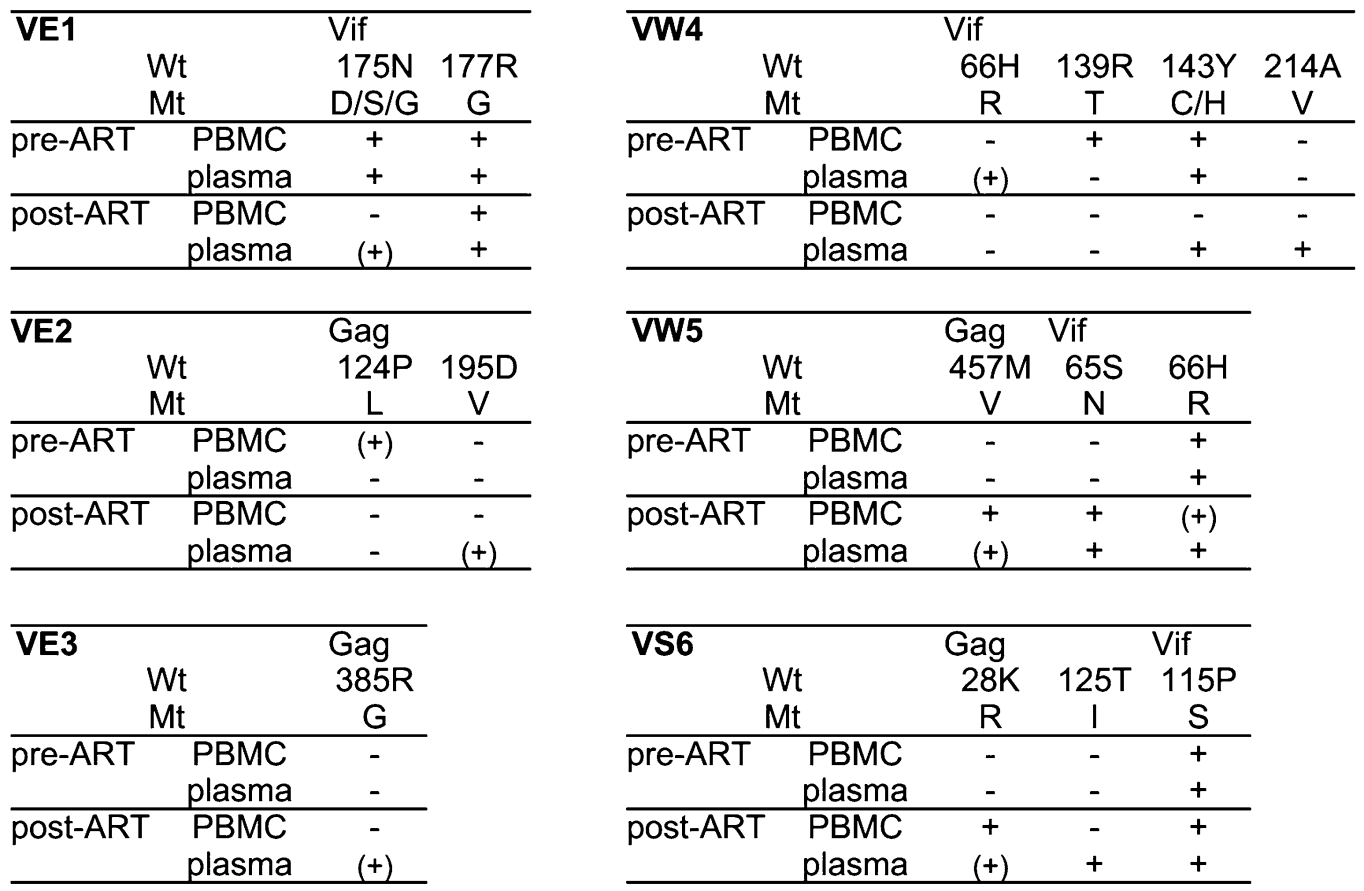
Non-synonymous *gag* and *vif* mutations in the vaccinated macaques. Viral *gag* and *vif* cDNAs were amplified from pre-ART and post-ART CD8^-^ cells-derived SIVs obtained at in vitro viral suppression assay and subjected to sequence analyses. Viral *gag* and *vif* cDNAs were also amplified from plasma-derived RNAs at weeks 12 and 34 and subjected to sequence analyses. + indicates that the non-synonymous mutation resulting in amino acid substitution was detected predominantly or equivalently with the wild-type sequence. (+) indicates that the wild-type sequence was predominant while the non-synonymous mutation was subdominantly detected.

There were some discrepancies between anti-pre-ART SIV and anti-post-ART SIV efficacy of CD8^+^ cells as expected. Analysis found some difference in viral *gag* and *vif* sequences between individual pre-ART and post-ART SIVs obtained from the vaccinated macaque PBMCs for in vitro viral suppression assay (Fig. 7). Additional non-synonymous mutations in post-ART SIV *gag* were detected in macaques VW5 (GagM457V) and VS6 (GagK28R). Especially in the latter macaque VS6, Gag_20–34_ peptide-specific CD8^+^ T-cell responses were detected, and CD8^+^ cells under ART (at week 27) showed much lower anti-post-ART SIV efficacy compared to anti-pre-ART SIV efficacy, suggesting that the GagK28R mutation may be responsible for the difference between anti-pre-ART SIV and anti-post-ART SIV efficacy of CD8^+^ cells.

### Correlation analyses between in vitro anti-SIV efficacy of CD8^+^ cells and plasma viral load in the vaccinated macaques

**Figure 8:**
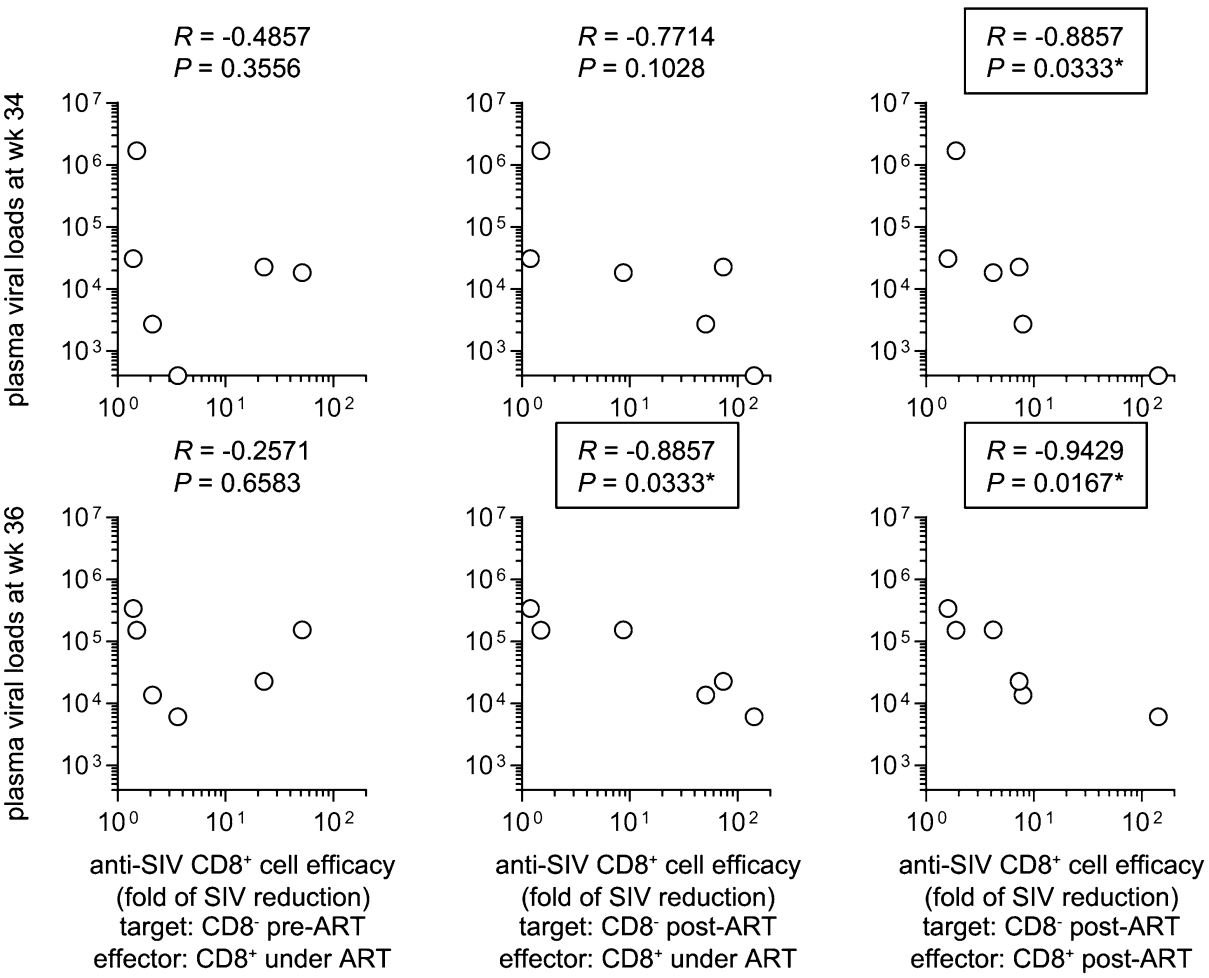
Correlation analyses between anti-SIV CD8^+^ cell efficacy and plasma viral loads in the vaccinated animals. Correlation analysis between anti-pre-ART SIV efficacy of CD8^+^ cells under ART (left panels) or anti-post-ART SIV efficacy of CD8^+^ cells under ART (middle panels) or post-ART (right panels) and viral loads at week 34 (upper panels) or 36 (lower panels) post-infection.

Finally, we examined correlation of in vitro anti-SIV CD8^+^ cell efficacy with plasma viral loads at weeks 34 and 36 (2 and 4 weeks after ART cessation) in Group V animals. There was no significant correlation between these plasma viral loads after ART and anti-pre-ART SIV efficacy of CD8^+^ cells under ART (Fig. 8). In contrast, anti-post-ART SIV efficacy of CD8^+^ cells post-ART was inversely correlated with plasma viral loads at weeks 34 (*R* = − 0.8857, *P* = 0.0333 by Spearman test) and 36 (*R* = − 0.9429, *P* = 0.0167), respectively (Fig. 8). Anti-post-ART SIV efficacy of CD8^+^ cells under ART exhibited a non-significant trend of association with lower viral load at week 34 (*R* = − 0.7714, *P* = 0.1028) but a significant inverse correlation with plasma viral load at week 36 (*R* = − 0.8857, *P* = 0.0333) (Fig. 8).

## Discussion

Prophylactic vaccines which are administered to naive (unvaccinated) individuals can dominantly induce vaccine antigen-specific CD8^+^ T-cell responses. In contrast, therapeutic vaccines are supposed to be administered to individuals already primed by HIV infection. Some HIV-infected individuals have MHC/HLA (human leukocyte antigen) class I genotypes associated with predominant Gag/Vif-specific CD8^+^ T-cell response but others have those with predominant CD8^+^ T-cell responses targeting non-Gag/Vif antigens. It is important to define the patterns of CD8^+^ T-cell responses after therapeutic vaccination in the latter cases as well as the former. In the present study, we examined the impact of therapeutic SeV-Gag/Vif vaccination under ART on SIV antigen-specific CD8^+^ T-cell responses in SIVmac239-infected rhesus macaques. In the three Group V macaques possessing MHC-I haplotype W/S associated with predominant Gag/Vif-specific CD8^+^ T-cell responses, Gag/Vif-specific CD8^+^ T-cell responses were enhanced after SeV-Gag/Vif vaccination and remained predominant after ART cessation (at week 34). In the remaining three Group V macaques possessing MHC-I haplotype E associated with predominant non-Gag/Vif antigen-specific CD8^+^ T-cell responses, Gag/Vif-specific CD8^+^ T-cell responses were enhanced to become predominant after SeV-Gag/Vif vaccination and remained predominant even after ART cessation (at week 34), although Nef/Env-specific CD8^+^ T-cell responses were predominant post-ART in the E-positive Group N macaques. These results indicate that therapeutic SeV-Gag/Vif vaccination under ART can enhance even subdominant Gag/Vif-specific CD8^+^ T-cell responses and change the CD8^+^ T-cell immunodominance.

Early diagnosis of HIV infection is not easy, and most HIV-infected individuals start ART after the viremia setpoint. We then began ART in macaques at week 12 post-infection, after the viremia setpoint with induction of SIV-specific T-cell responses, in the present study. The first therapeutic vaccination was performed 3 months after the ART initiation, when SIV-specific CD8^+^ T-cell responses were largely reduced. This protocol is considered to be good for the analysis of changes in immunodominance by therapeutic vaccination described above. However, CD8^+^ T-cell escape SIV variants are expected to appear during the 12 weeks before the ART initiation. We thus examined efficacy of CD8^+^ cells to suppress replication of autologous PBMC-derived SIVs as described in the Materials and Methods, although it is difficult to exactly adjust effector-target ratios in the analysis. Analysis of the ability of CD8^+^ cells to suppress replication of autologous PBMC-derived SIVs revealed an increase in in vitro anti-SIV efficacy of CD8^+^ cells by the first SeV-Gag/Vif vaccination under ART, confirming that therapeutic SeV-Gag/Vif vaccination can augment anti-SIV efficacy of CD8^+^ cells. The anti-pre-ART SIV efficacy of CD8^+^ cells at week 27 (1 week after the first SeV-Gag/Vif vaccination) was significantly correlated with Gag-specific CD8^+^ T-cell responses at week 27, implying that induction of Gag-specific CD8^+^ T-cell responses by therapeutic vaccination can result in augmentation of anti-SIV efficacy of CD8^+^ cells. The vaccinated animals showed rebound viremia after ART cession, indicating that these augmented CD8^+^ cells are not able to contain SIV replication without ART. To increase the possibility of controlling viremia rebound after ART cession, anti-SIV IgGs were administered to help CD8^+^ T cell-based viremia control, but the administered anti-SIV IgGs were considered to be insufficient for showing any efficacy in our results. At week 34 (2 weeks after ART cessation), however, Gag-specific CD8^+^ T-cell responses were correlated with anti-post-ART SIV efficacy of CD8^+^ cells, which was inversely correlated with rebound plasma viral loads. Thus, augmentation of anti-SIV efficacy of CD8^+^ cells by vaccine-mediated Gag-specific CD8^+^ T-cell induction may contribute to more stable viral control under ART.

Current attempts of therapeutic vaccination inducing SIV-specific T-cell responses under ART in macaques have shown promising results, delay or control of viremia rebound after ART cession^32,33^. However, our experiments did not exhibit significant effect on viremia rebound after ART. One possible important difference between these previous studies and the present study may be the time point of ART initiation. ART was initiated in a week post-infection in the previous studies but at week 12 in the present study. It is speculated that CD8^+^ T-cell escape SIV variants appear during the 12 weeks post-infection^14,34^, which can disturb the efficacy of wild-type antigen-specific CD8^+^ T-cell responses induced by vaccination against viral replication. Indeed, several *gag*/*vif* mutations were detected before the ART initiation at week 12. Therefore, induction of CD8^+^ T-cell responses targeting Gag, the most conserved HIV/SIV antigen, by therapeutic vaccination could have greater impact on viral control as indicated by our results. In addition, SIV-specific T-cell responses induced before ART initiation post-infection can affect dominant/subdominant T-cell responses induced by therapeutic vaccination as described above. These results would provide insights into the development of therapeutic vaccination under ART initiated after the viremia setpoint.

In summary, the present study showed that therapeutic SeV-Gag/Vif vaccination under ART initiated after the viremia setpoint can enhance subdominant as well as predominant Gag/Vif-specific CD8^+^ T-cell responses in SIV-infected rhesus macaques. Our results indicate that induction of Gag-specific CD8^+^ T-cell responses by therapeutic vaccination can augment anti-virus efficacy of CD8^+^ cells, which may be insufficient for functional cure but result in more stable viral control under ART.

## Materials and methods

### Animal experiments

Animal experiments were carried out in the Institute for Frontier Life and Medical Sciences, Kyoto University after approval by the Committee on the Ethics of Animal Experiments in Kyoto University (permission number: R10-05 and R11-05) under the guidelines for animal experiments in accordance with the Guidelines for Proper Conduct of Animal Experiments established by Science Council of Japan (https://www.scj.go.jp/ja/info/kohyo/pdf/kohyo-20-k16-2e.pdf). The experiments were in accordance with Weatherall report for the use of non-human primates in research (https://royalsociety.org/topics-policy/publications/2006/weatherall-report/). Blood collection, virus inoculation, vaccination, and antibody administration were performed under ketamine anesthesia.

Two sets of similar experiments (Exp. 1 and Exp. 2) were performed using in total twelve Burmese rhesus macaques (*Macaca mulatta*) as shown in Table 1. We used three groups of rhesus macaques possessing the MHC-I haplotypes *90-010-Ie* (E) (n = 6), *89-075-Iw* (W) (n = 4), and *91-010-Is* (S) (n = 2), respectively^35–37^. These twelve animals were intravenously inoculated with 1,000 50% tissue culture infective doses (TCID_50_) of SIVmac239^38^. Viral loads and T-cell responses pre-ART in macaques NE1, NE2, NW4, NS6, VE1, VE2, VW4, and VS6 were described before^36^. All of these macaques received ART from week 12 to 32 post-infection. During this period, food including Zidovudine/Lamivudine (AZT/3TC), Tenofovir disoproxil fumarate (TDF), and Lopinavir/Ritonavir (LPV/r) was given to the animals as described before^39^. The daily dosages were 300 mg of AZT and 150 mg of 3TC (Combivir, GlaxoSmithKline), 300 mg of TDF (Viread, Japan Tobacco) and 400 mg of Lopinavir and 100 mg of Ritonavir (Kaletra, Abbott Laboratories). Twelve animals were divided into Groups N (unvaccinated, n = 6) and V (vaccinated, n = 6). Group V macaques (three E^+^, two W^+^ and one S^+^) were intranasally immunized with 6 × 10^9^ cell infectious units (CIU) of F-deleted replication-defective Sendai virus (SeV) vectors expressing SIVmac239 Gag (SeV-Gag) and Vif (SeV-Vif)^28–30^, respectively, at weeks 26 and 32 (2 days before ART cessation). Two Group N (NE3 and NW5) and all six Group V macaques were intravenously administered with 160 mg/4 ml of polyclonal anti-SIV IgGs at 2 days before and 4 days after ART cessation. The polyclonal anti-SIV IgG was purified from plasma of SIVmac239-infected macaques (2,520 mg IgG from approximately 380 ml of plasma) as described before^40^.

### Analysis of antigen-specific CD8^+^ T-cell responses

We measured SIV antigen-specific CD8^+^ T-cell frequencies by flow cytometric analysis of IFN-γ induction after specific stimulation as described previously^41^. Peripheral blood mononuclear cells (PBMCs) were cocultured with autologous herpesvirus papio-immortalized B-lymphoblastoid cell lines (B-LCL) pulsed with peptide pools (at a final concentration of 1 μM for each peptides) using panels of overlapping peptides spanning the entire SIVmac239 Gag, Pol, Vif, Vpx, Vpr, Tat, Rev, Env and Nef amino acid sequences in the presence of GolgiStop (monensin; BD) for 6 h. Cells were harvested and intracellular IFN-γ staining was performed using a Cytofix-Cytoperm kit (Becton Dickinson) and fluorescein isothiocyanate-conjugated anti-human CD4 (BD), peridinin chlorophyll protein-conjugated anti-human CD8 (BD), allophycocyanin-conjugated anti-human CD3 (BD) and phycoerythrin-conjugated anti-human IFN-γ monoclonal antibodies (BioLegend) (Fig. 2a). Specific CD8^+^ T-cell frequencies were calculated by subtracting nonspecific IFN-γ^+^ CD8^+^ T-cell frequencies from those after peptide-specific stimulation. Specific CD8^+^ T-cell frequencies of less than 0.05% per CD8^+^ T cells were considered negative.

### In vitro viral suppression assay

We examined the ability of CD8^+^ cells to suppress replication of SIVs derived from autologous CD8^−^ cells. PBMCs were separated into CD8^+^ and CD8^−^ cells by using Macs CD8 MicroBeads (Miltenyi Biotec). CD8^−^ cells selected from PBMCs at weeks 10 (before ART initiation) and 34 (after ART cessation) were used for targets pre-ART and post-ART, respectively. These target cells (10^5^ cells in cases with > 5 × 10^4^ copies/ml of plasma viral load [in NE1, NE3, NW4, NW5, VE1, VE3, VW5, and VS6 for pre-ART and VW5 for post-ART] or 2 × 10^5^ cells in cases with < 5 × 10^4^ copies/ml of plasma viral load) [in others]) were cultured in the presence of 2 μg/ml of phytohemagglutinin L and 20 IU/ml of recombinant human interleukin-2 (Roche Diagnostics) for two days, and then, cocultured with 5 × 10^4^ CD8^+^ cells (effector) selected from PBMCs at weeks 10 (pre-ART), 27 (under ART), and 34 (post-ART), respectively. After 6 days of culture, the culture supernatants were harvested and subjected to analysis of SIV p27 concentrations by enzyme-linked immunosorbent assay (ELISA) using SIV p27 Antigen Capture Assay (Advanced Bioscience Laboratories). If the non-CD8 culture (the culture without CD8^+^ cells) had lower than 100 ng/ml of p27, the culture supernatants on day 8 (VE2 pre-ART, VE3 post-ART, VW4 post-ART, and VS6 pre-ART) or 10 (VE2 post-ART) were used for the analysis. Because SIV replication kinetics are considered to be different among the cultures of target cells derived from individual SIV-infected macaque PBMCs, it is difficult to exactly adjust effector-target ratios in all the in vitro viral suppression assay by using limited available samples. Then, as described above, the viral suppression was analyzed under the condition that the individual negative control target cultures without CD8^+^ cells were adjusted to produce more than 100 ng/ml of p27 not on day 4 but on day 6 or later. The ratios of p27 concentrations in the supernatants from the coculture to those without CD8^+^ cells are shown as fold of reduction (in vitro anti-SIV efficacy)^42^.

### Analysis of viral *gag* and *vif* sequences

Viral RNAs were extracted using the high pure viral RNA kit (Roche Diagnostics) from SIVs pre-ART and post-ART, the culture supernatants of CD8^-^ cells derived from PBMCs at weeks 10 and 34 post-infection in individual animals. Viral RNAs were also extracted from plasma samples at weeks 12 and 34. Viral *gag* and *vif* cDNAs were amplified from these RNAs by reverse transcription and nested PCR using the PrimeScript one-step RT-PCR kit version 2 (Takara) and KOD-Plus version 2 (Toyobo), and subjected to direct sequencing by using dye terminator chemistry and an automated DNA sequencer (Applied Biosystems) as described before^37^.

### Statistical analysis

Statistical analysis was performed using Prism software version 6.0d (GraphPad Software Inc.) with significance levels set at a *P* value of less than 0.05. Specific CD8^+^ T-cell frequencies, in vitro anti-SIV efficacy and plasma viral loads were log transformed. Comparison was performed by Mann–Whitney *U* test. Correlation analysis was performed by Spearman test.
